# Sexual violence and associated factors among women of reproductive age in Rwanda: a 2020 nationwide cross-sectional survey

**DOI:** 10.1186/s13690-023-01109-z

**Published:** 2023-06-19

**Authors:** Lilian Nuwabaine, Joseph Kawuki, Earnest Amwiine, John Baptist Asiimwe, Quraish Sserwanja, Ghislaine Gatasi, Elorm Donkor, Humphrey Atwijukiire

**Affiliations:** 1 School of Nursing and Midwifery, Aga Khan University, Kampala, Uganda; 2 Jockey Club School of Public Health and Primary Care, Faculty of Medicine, The Chinese University of Hong Kong, Hong Kong, China; 3 Faculty of Medicine, Mbarara University of Science & Technology, Mbarara, Uganda; 4 Programmes Department, Relief International, Khartoum, Sudan; 5 Key Laboratory of Environmental Medicine Engineering, School of Public Health, Southeast University, 210009 Nanjing, Jiangsu Province China; 6 Department of Nursing, Bishop Stuart University, Mbarara, Uganda

**Keywords:** Sexual violence, Women, Sexual abuse, Rwanda

## Abstract

**Background:**

Sexual violence against women is a global public health issue with both short- and long-term effects on the physical and mental health of women. This study aimed to determine the prevalence of sexual violence and its associated factors among women of reproductive age in Rwanda.

**Methods:**

We used secondary data from the 2020 Rwanda Demographic and Health Survey of 1,700 participants, who were selected using multistage stratified sampling. Multivariable logistic regression was conducted to explore factors associated with sexual violence using SPSS (version 25).

**Results:**

Of the 1,700 women of reproductive age, 12.4% (95%CI: 11.0–14.1) had experienced sexual violence. Justified beating (AOR = 1.34, 95%CI: 1.16–1.65), not having health insurance (AOR = 1.46, 95%CI: 1.26–2.40), not being involved in healthcare decision-making (AOR = 1.64, 95%CI: 1.99–2.70), having a husband/partner with primary (AOR = 1.70, 95%CI: 5.47–6.21) or no education (AOR = 1.84, 95%CI: 1.21–3.37), as well as having a husband/partner who sometimes (AOR = 3.37, 95%CI: 1.56–7.30) or often (AOR = 12.87, 95%CI: 5.64–29.38) gets drunk were positively associated with sexual violence. However, women from male-headed households (AOR = 0.52, 95%CI: 0.29–0.92) were less likely to experience sexual violence.

**Conclusions:**

There is a need to demystify negative culturally-rooted beliefs favouring sexual violence, such as justified beating, as well as increase efforts to promote women’s empowerment and healthcare access. Moreover, engaging men in anti-sexual violence strategies is paramount to addressing male-related issues that expose women to sexual violence.

**Supplementary Information:**

The online version contains supplementary material available at 10.1186/s13690-023-01109-z.

**Table:** 

**Text box 1. Contributions to the literature**
• Sexual violence is one of the commonest forms of violence experienced by women and negatively impacts both the victims and their families.
• Our study revealed a substantial prevalence of sexual violence among women of reproductive age in Rwanda which is although lower than the overall worldwide prevalence.
• There is a need to demystify negative culturally-rooted beliefs fueling not only sexual violence but also physical violence, as well as efforts to promote women’s empowerment to enable them to make healthier decisions.
• Engaging men in anti-sexual violence strategies is paramount in addressing male-related issues that expose women to violence.

## Background

Sexual violence is one of the most common forms of violence experienced by women [1, 2]. Sexual violence refers to any sexual act or attempts to obtain sexual act against an individual’s will, unwanted sexual advances and comments that may be directed against anyone’s sexuality regardless of who the perpetrator is and where it is happening [2]. Sexual violence manifests in different forms like sexual harassment, completed rape, attempted rape, degrading or humiliating sexual acts, bad touches and unwanted sexual comments [3]. Males are reported to be the most common sexual violence perpetrators, including acquaintances, family members, and intimate partners like boyfriends and husbands and very few occurrences are attributable to strangers [4–6].

Multiple studies have shown that sexual violence negatively impacts both the victims and their families [7–11]. Women who experience sexual violence are at risk of contracting Human Immunodeficiency Virus (HIV) and other sexually transmitted infections, unwanted pregnancies, stigmatization, family break-ups, depression and resorting to alcohol and drugs as a coping mechanism [12]. There are instances where this violence also results in physical harm [8, 10, 11, 13]. However, these effects were found to be more pronounced among pregnant women due to their vulnerable state [14]. Studies show that expectant women who are victims of sexual violence are at higher risk of pregnancy-related complications such as preterm birth, low birth weight, and delayed prenatal care [15, 16].

Sexual violence towards women is a major violation of human rights and a global public health challenge with mental and physical effects on the well-being of women and their children [17–19]. The World Health Organization (WHO) report shows that 27% of women aged 15–49 experience either sexual or physical violence in their lifetime [2]. Other reports show that at least one in every three women experiences sexual violence in their lifetime [2, 17, 20, 21]. A review of databases from 195 countries worldwide revealed an overall prevalence of sexual violence against women at 35.6% [22].

A systematic analysis of studies in sub-Saharan Africa shows that the overall prevalence of sexual violence against women is 18.7% [20]. South Africa still reports the highest prevalence rates of sexual violence against women aged 18–49 years at 37.9% [23]. In East African countries like Kenya and Uganda, lifetime sexual violence stands at 20.5% and 24.3%, respectively [24, 25]. In those countries, the prevalence of sexual violence has been demonstrated to also be high among pregnant women, at 34.8% and 36.1%, respectively [19, 26].

Various studies done in sub-Saharan Africa show that sexual violence, just like other forms of violence against women, is influenced by multiple factors [27–31]. Some of the factors include individual factors like age and education level, demographic characteristics like living in rural areas, social and contextual factors like secretive culture, inadequate religious beliefs, and gender inequalities, and finally, need factors like relying on husbands for basic survival [27–31]. Additional factors such as witnessing family sexual violence during childhood, drug abuse, difficulties in effective communication among couples, marital discord, male controlling behaviour, and community norms were found to be associated with sexual violence [19, 32]. Current pregnancy status has also previously been identified as one of the associated factors of sexual violence [33]. A systematic review found a worldwide prevalence of sexual violence among pregnant women of 31% [34]. Another systematic review and meta-analysis of the worldwide prevalence of intimate partner violence (IPV) among pregnant women revealed that sexual violence accounted for 5.5% of IPV cases [35].

Sexual violence among women and its predictors in Rwanda have been barely explored, with the existing few studies focusing on pregnant women [36, 37] and female sex workers [38]. With this scanty literature, it creates a gap in the formulation and implementation of practical interventions in addressing this public health issue. Therefore, this study aimed at assessing the prevalence of sexual violence and its associated factors among women of reproductive age in Rwanda, using the recent 2020 Rwanda Demographic and Health survey. This information would help in guiding policy formulations and interventions aimed at addressing sexual violence.

## Methods

### Study sampling and participants

The 2019–20 Rwanda Demographic Survey (RDHS) data was used for this analysis. The RDHS employed a two-stage sample design; with the first stage involving cluster selection consisting of enumeration areas (EAs) and the second stage involving systematic sampling of households in all the selected EAs leading to a total of 13,005 households [39]. The data used in this analysis were particularly from the household and domestic violence questionnaires.

The 2019–2020 RDHS data were collected between November 2019 and July 2020 [39]. RDHS-eligible participants were women aged 15–49 years who were either permanent residents of the selected households or visitors who stayed in the household the night before the survey. In two-thirds of the total households, one eligible woman per household was randomly selected to respond to the domestic violence module as part of the individual interview, and the module was not implemented if privacy could not be assured. Of the 14,675 women aged 15–49 interviewed in the RDHS, 7,402 were selected to respond to the domestic violence questions. This analysis, however, only included 1,700 women who had full responses for sexual violence items/ questions.

## Variables

### Dependent variables

The study outcome variable was the history of exposure to sexual violence among women in Rwanda, and it was a binary variable coded yes or no. During the survey, information was collected about the ever-married women’s lifetime experiences of any form of sexual violence (by their current or previous partner) by asking about the experience of the following: anyone physically forcing you to have sexual intercourse with him or even when you do not want to; physically forcing you to perform any other sexual acts you do not want to; forcing you with threats or in any other way to perform sexual acts you do not want to [39].

### Explanatory variables

Determinants of sexual violence were included based on the available literature and data [27–31, 37, 40, 41]. Twenty-one (21) variables were considered and of these, two were community-level factors that included place of residence (categorized into rural and urban), and region of residence (Kigali, South, West, East, and North). Seven household-level factors included household size (less than six and six and above), sex of household head (male and female), husband/partner’s educational level, husband/partner’s age, husband/partner’s working status (yes or no), husband/partner’s frequency of being drunk (never, sometimes, and often), and wealth index (categorized into five quintiles that ranged from the poorest to the richest quintile). Twelve individual-level factors were also considered in the analysis, including; age (15–24, 25–34, 35–44, and 45–49 years), working status (yes or no), justified beating (yes or no), parity (4 and less and above 4) educational level (no education, primary, secondary, and tertiary), health insurance (yes and no), religion (catholic, protestant, Adventist, Moslem and others), health care decision-making (yes and no), exposure to radio, newspapers, and television (yes and no), and economic empowerment (high, medium, low, and no empowerment). The wealth index was calculated by RDHS from information on household asset ownership using Principal Component Analysis. In addition, the answer to the husband/partner’s frequency of getting drunk was the woman’s subjective judgment, and justified beating was determined by asking women whether they thought it was okay to be beaten by their partner for a specific reason [39].

### Statistical analysis

To account for the unequal probability sampling in different strata and ensure the representativeness of the study results, DHS sample weights were applied [42, 43]. Analysis was conducted using SPSS (version 25) software – complex samples package, which accounted for the multistage sample design inherent in the RDHS dataset by incorporating: individual sample weight, sample strata for sampling errors/design, and cluster number in the analysis plan [39, 43, 44]. Frequency distributions were used to describe the background characteristics of the respondents. Bivariable logistic regression was then conducted to assess the association of each predictor variable with sexual violence, and crude odds ratio (COR), 95% confidence interval (CI) and p-values were presented. Independent variables found significant at a p-value < 0.25 were then included in the multivariable model. Additionally, other variables reported to have a significant association with sexual violence in previous studies, regardless of their significance on bivariable analysis, were also included. Adjusted odds ratios (AOR), 95%CI and p-values were obtained and presented, with a statistical significance level set at p-value < 0.05. All predictor variables in the model were assessed for multi-collinearity, which was considered present if a variable had a variance inflation factor (VIF) greater than 10 [42]. However, none of the variables had a VIF above 3.

## Results

### Characteristics of participants

A total of 1,700 participants were included in this analysis (Table 1). The majority were 25–44 years of age (78.5%), attained a primary level of education (64.4%), working (77.5%) and had parity of not more than 4 children (77.6%). Additionally, 50.7% justified being beaten by their husbands, 83.1% had health insurance, 84.5% were rural residents, 70.9% were from households of less than 6 members, and 85.3% were from male-headed households. Moreover, 77% were exposed to radio, while 60.5% and 81.8% had no exposure to television or newspapers, respectively, 81.2% had healthcare decision-making, and 93.3% had working husbands with primary education (69%). Regarding the experience of sexual violence, 211 (12.4% 95%CI: 11.0–14.1) had ever been sexually abused.

**Table 1:** Background characteristics of Rwandan women aged 15 to 49 years as per the 2020 Rwanda Demographic Health Survey

Characteristics	Frequency (%), N = 1,700
Age	
45–49	147(8.6)
35–44	589(34.7)
25–34	745(43.8)
15–24	219(12.9)
**Education level**	
Tertiary	73(4.3)
Secondary	313(18.4)
Primary	1095(64.4)
No education	218(12.8)
**Working status**	
Working	1317(77.5)
Not working	382(22.5)
**Parity**	
Above 4	380(22.4)
4 and less	1319(77.6)
**Justified beating**	
No	838(49.3)
Yes	861(50.7)
**Health insurance**	
Yes	1412(83.1)
No	287(16.9)
**Religion**	
Catholic	613(36.1)
Protestant	801(47.1)
Adventist	231(13.6)
Muslim	25(1.4)
Others	29(1.7)
**Wealth index**	
Richest	305(18.0)
Richer	376(22.1)
Middle	330(19.4)
Poorer	347(20.4)
Poorest	342(20.1)
**Residence**	
Urban	264(15.5)
Rural	1436(84.5)
**Region**	
Kigali	226(13.3)
West	378(22.2)
East	446(26.3)
North	281(16.5)
South	368(21.6)
**Household size**	
Less than 6	1204(70.9)
6 and above	495(29.1)
**Sex of household head**	
Female	250(14.7)
Male	1450(85.3)
**Exposure to radio**	
Yes	1308(77.0)
No	392(23.0)
**Exposure to television**	
Yes	671(39.5)
No	1029(60.5)
**Exposure to newspapers**	
Yes	309(18.2)
No	1391(81.8)
**Economic empowerment**	
High	426(25.1)
Medium	653(38.4)
Low	461(27.1)
No	160(9.4)
**Healthcare decision-making**	
Yes	1380(81.2)
No	320(18.8)
**Husband/partner’s age**	
45–49	365(21.5)
35–44	651(38.3)
25–34	606(35.7)
15–24	77(4.5)
**Husband/partner’s education**	
Tertiary	80(4.7)
Secondary	211(12.4)
Primary	1173(69.0)
No education	236(13.9)
Husband/partner’s frequency of getting drunk *	
Never	245(14.4)
Sometimes	633(37.3)
Often	150(8.9)
**Husband/partner’s working status**	
Yes	1585(93.3)
No	114(6.7)
**Sexual violence**	
No	1489(87.6)
Yes	**211(12.4), (95%CI: 11.0–14.1)**

* = 671 missing values

### Factors associated with sexual violence among women of reproductive age

The results of the bivariable analysis are detailed in Table 2, with factors having an independent significant association with sexual violence highlighted. At the multivariable analysis level, the factors found significantly associated with sexual violence were justified beating, health insurance, sex of household head, healthcare decision-making, husband/partner’s education, and husband’s frequency of getting drunk (Table 2).

Women who justified beating (AOR = 1.34, 95%CI: 1.16–1.65) had higher odds of experiencing sexual violence compared to those who did not, similar to women with no health insurance (AOR = 1.46, 95%CI: 1.26–2.40) who also had higher odds of being sexually abused compared to those with health insurance. Compared to women involved in healthcare decision-making, those not involved in healthcare decision-making (AOR = 1.64, 95%CI: 1.99–2.70) had higher odds of being sexually abused. Similarly, women with husband/partner of no education (AOR = 1.84, 95%CI: 1.21–3.37) and primary education (AOR = 1.70, 95%CI: 5.47–6.21) had higher odds of experiencing sexual violence compared to those whose husbands had tertiary education. Moreover, women with husbands who often (AOR = 12.87, 95%CI: 5.64–29.38) and sometimes (AOR = 3.37, 95%CI: 1.56–7.30) got drunk also had higher odds of being sexually abused, compared with those with husbands who never get drunk. However, women from male-headed households (AOR = 0.52, 95%CI: 0.29–0.92) had less odds of experiencing sexual violence compared to their counterparts from female-headed households.

**Table 2:** Factors associated with sexual violence among women in Rwanda as per the 2020 RDHS

Characteristics	Crude odds ratio, COR (95% CI)	p-value*	Adjusted odds ratio, AOR (95% CI)	p-value**
**Age**		0.270		0.781
45–49	1		1	
35–44	0.94(0.48–1.84)		0.76(0.36–1.61)	
25–34	0.83(0.42–1.63)		0.63(0.25–1.59)	
15–24	050(0.21–1.16)		0.53(0.14–1.97)	
**Education level**		**0.135**		0.123
Tertiary	1		1	
Secondary	3.37(0.95–12.03)		2.78(0.53–14.68)	
Primary	4.01(1.24–13.04)		1.35(0.24–7.73)	
No education	3.87(1.12–13.33)		1.15(0.19–7.08)	
**Working status**		0.337		0.190
Working	1		1	
Not working	0.80(0.50–1.27)		0.68(0.38–1.22)	
**Parity**		0.270		0.390
Above 4	1		1	
4 and less	0.82(0.57–1.17)		0.81(0.50–1.31)	
**Justified beating**		**0.031**		**0.038**
No	1		1	
Yes	1.46(1.04–2.05)		**1.34(1.16–1.65)**	
**Health insurance**		**0.005**		**0.043**
Yes	1		1	
No	1.94(1.22–3.09)		**1.46(1.26–2.40)**	
**Religion**		0.552		0.309
Catholic	1		1	
Protestant	1.33(0.90–1.97)		1.40(0.87–2.26)	
Adventist	1.46(0.82–2.61)		1.56(0.83–2.94)	
Muslim	1.64(0.57–4.72)		3.18(0.86–11.82)	
Others	1.05(0.29–3.77)		1.13(0.24–5.48)	
**Wealth index**		**0.089**		0.253
Richest	1		1	
Richer	1.35(0.63–2.88)		0.66(0.29–1.51)	
Middle	2.24(1.10–4.55)		1.41(0.57–3.53)	
Poorer	2.23(1.132–4.41)		1.07(0.45–2.57)	
Poorest	1.93(0.96–3.86)		0.82(0.33–2.01)	
**Residence**		0.538		0.190
Urban	1		1	
Rural	1.21(0.66–2.24)		0.68(0.38–1.21)	
**Region**		0.825		0.801
Kigali	1		1	
West	1.18(0.58–2.42)		0.94(0.45–1.94)	
East	0.88(0.42–1.84)		0.82(0.39–1.71)	
North	1.08(0.52–2.25)		0.87(0.34–2.21)	
South	1.08(0.52–2.26)		0.66(0.30–1.45)	
**Household size**		0.331		0.801
Less than 6	1		1	
6 and above	0.82(0.55–1.22)		1.07(0.62–1.85)	
**Sex of household head**		0.335		**0.024**
Female	1		1	
Male	0.77(0.45–1.31)		**0.52(0.29–0.92)**	
**Exposure to radio**		**0.161**		0.171
Yes	1		1	
No	1.30(0.90–1.87)		1.38(0.87–2.17)	
**Exposure to television**		0.375		0.814
Yes	1		1	
No	1.19(0.81–1.75)		0.94(0.58–1.54)	
**Exposure to newspapers**		0.496		0.701
Yes	1		1	
No	1.18(0.73–1.92)		0.88(0.45–1.72)	
**Economic empowerment**		0.283		0.124
High	1		1	
Medium	1.20(0.78–1.84)		1.63(0.93–2.84)	
Low	0.75(0.43–1.29)		0.86(0.43–1.71)	
No	1.05(0.60–1.86)		1.15(0.50–2.62)	
**Healthcare decision-making**		**0.130**		**0.045**
Yes	1		1	
No	1.34(0.92–1.95)		**1.64(1.99–2.70)**	
**Husband/partner’s age**		0.812		0.696
45–49	1		1	
35–44	0.99(0.63–1.58)		1.40(0.76–2.55)	
25–34	0.87(0.55–1.36)		1.24(0.64–2.43)	
15–24	0.70(0.25–1.98)		0.86(0.17–4.27)	
**Husband/partner’s education**		**0.114**		**0.026**
Tertiary	1		1	
Secondary	1.23(0.37–4.12)		0.69(0.18–2.66)	
Primary	2.76(0.91–8.37)		**1.70(1.47–6.21)**	
No education	2.05(0.63–6.62)		**1.84(1.21–3.37)**	
**Husband/partner’s frequency of getting drunk**		**<0.001**		**<0.001**
Never	1		1	
Sometimes	3.18(1.51–6.68)		**3.37(1.56–7.30)**	
Often	11.69(5.34–25.58)		**12.87(5.64–29.38)**	
**Husband/partner’s working status**		**0.110**		0.990
Yes	1		1	
No	1.58(0.90–2.77)		0.99(0.41–2.40)	

**Bold** = significant, *= significant at 0.25, **= significant at 0.05, RDHS = Rwanda demographic health survey

## Discussion

This study assessed the prevalence of sexual violence and associated factors among women of reproductive age in Rwanda. The results showed that about 12% of women in Rwanda had experienced sexual violence. This prevalence is lower than that reported in other East African countries like Uganda (24.3%) and Kenya (20.5%) [24, 25], and that reported in South Africa (24.9%) [23]. This could be explained by differences in socio-demographic characteristics, cultural norms, and beliefs, as well as weak judicial systems that put victims at risk and higher incidences of sexual abuse [45]. Studies have shown that weak judicial systems to hold perpetrators accountable and bad social norms that fuel sexual violence are great players in the incidence of sexual violence [46, 47]. The lower observed prevalence may also be due to the fact that Rwanda set up initiatives to uplift the rights of women post-genocide [48]. However, there is a need for streamlining and strengthening policies and laws aimed at empowering women, engaging men who are the likely perpetrators and creating gender equality to protect women from existing sexual injustices.

In this study, women who justified being beaten by their husbands were more likely to experience sexual violence than those who did not justify beating. This concurs with results from other studies in Nigeria and Uganda that reported a positive link between justified beating and sexual violence as well as physical and emotional violence [49, 50]. This goes in hand with social norms that empower husbands with the right to use violence against their women and women that are so protective of their family image rather than their health challenges [46]. Communities with such negative cultural beliefs usually do not consider sexual violence as a type of violation, and this kind of mindset denies women self-confidence and makes them vulnerable to many forms of abuse by men [51]. Therefore, sensitization of men, who are the likely perpetrators of sexual violence focusing on debunking such negative cultural beliefs is crucial in addressing the violence of all forms against women.

Not having health insurance as well as not being able to participate in healthcare decision-making were positively associated with sexual violence in this study. These findings are consistent with results from other studies conducted in Nigeria and the United States of America which showed that women with health insurance had an upper hand in decision-making about their health and are more likely to be informed about different health issues which is one of the fundamentals of woman empowerment [52]. This is supported by the fact that woman empowerment and sexual violence have a negative correlation according to *Shabnam et al.* [52]. Women who are empowered are more able to identify sexual risk behaviours and take appropriate decisions to lower the risk of being sexually violated [53, 54].

Women who lived in households headed by males were less likely to face sexual violence than those who lived in female-headed households. There is a dearth of literature comparing this observation. However, belonging to a male-headed household may provide protection since perpetrators could fear the man in the house. Additionally, this observation may be because power struggles may arise due to cultural perceptions and expectations of women being subordinate and accommodative to men’s actions. Thus, women heading families are more likely to be assertive resulting in more relational challenges that could increase the risks of sexual violence [52, 55].

Study findings indicate that women whose husbands had no, or primary education were more likely to be sexually violated compared to those with husbands/partners of tertiary education. This concurs with studies conducted in Zambia and South Asia that also reported a negative association between education level and sexual violence [56, 57]. This may be because partners with higher education tend to be aware of and value women’s rights, and are also more likely to know different grave effects of sexual violence [58]. Moreover, evidence shows an increase in sexual knowledge and a positive change in attitude towards violence following education and this would even be more pronounced if education is focused towards sexual practices [59]. Therefore, it is paramount that there is continuous community sensitization targeting men with a lower level of education and re-emphasizing to the middle and highly educated ones the dangers of sexual violence and other forms of violence.

This study found that women with husbands/partners who often or sometimes got drunk were more likely to be sexually abused compared to their counterparts with husbands who never get drunk. This is in line with studies in East Africa that also reported husbands’ drinking behaviour as a predictor of sexual abuse [25, 33]. This could be related to the unstable mental state and the aggressive behaviour that comes along with being drunk. Therefore, policies regulating alcohol drinking could put women at an advantage by reducing their risk of being sexually violated. Additionally, initiatives focusing on addressing other confounding factors to alcoholism, like personality traits, impulsivity, and hostility to women, that increase the likelihood of alcoholics committing sexual violence could be beneficial [60, 61].

## Strengths and Limitations

This study used the DHS dataset which comes from standardized data collection procedures, thus ensuring the internal and external validity of the results. In addition, we used the most recent nationally representative sample and weighted the data for analysis, and therefore, our results are generalized to all Rwandan women aged 15–49 years. However, the study had some limitations worth acknowledging. There is a possibility of information bias, as well as recall and interviewer biases since most data on the determinants were based on self-reporting, and respondents gave answers about events that occurred in the past. Moreover, the possibility of social desirability bias in answering sensitive questions about sexual violence can’t be overlooked, and this could have affected the true estimation of sexual violence prevalence. There was also a lack of data on other possible determinants of sexual violence such as drug and substance use, and missing data on some key outcomes was inevitable. Despite the above limitations, the study provides valuable information on the potential predictors of sexual violence among women in Rwanda.

## Conclusions

This study revealed a substantial prevalence of sexual violence among women of reproductive age in Rwanda, which is although lower than the overall worldwide prevalence. It however implies that sexual violence is still a major health challenge in Rwanda. In addition, several socio-demographic factors such as justified beating, health insurance, sex of household head, healthcare decision-making, partner’s education, and husband’s frequency of getting drunk were significantly associated with sexual violence against women. This highlights specific different areas worth consideration when rethinking policies to address sexual violence. There is a need to demystify negative culturally-rooted beliefs fueling not only sexual violence but also physical violence, as well as efforts to promote women’s empowerment to enable them to make healthier decisions. Moreover, engaging men in anti-sexual violence strategies is paramount in addressing male-related issues that expose women to violence in all its forms. A supplementary file 1 consisting of what is known, what the study adds and what the implications are for clinical practice, public health and/or research has been added.

## Electronic supplementary material

Below is the link to the electronic supplementary material.

Supplementary Material 1

## Data Availability

The data set used is openly available upon permission from the MEASURE DHS website (URL: https://www.dhsprogram.com/data/available-datasets.cfm). However, authors are not authorized to share this data set with the public but anyone interested in the data set can seek it with written permission from the MEASURE DHS website (URL: https://www.dhsprogram.com/data/available-datasets.cfm).

## Abbreviations

HIV: Human Immunodeficiency Syndrome
EA: Enumeration area
IPV: Intimate Partner Violence
AOR: Adjusted Odds Ratio
RDHS: Rwanda Demographic Health Survey
CI: Confidence Interval
WHO: World Health Organisation
DHS: Demographic Health Survey
VIF: Variance Inflation Factor
COR: Crude Odds Ratio
OR: Odds Ratio
SPSS: Statistical Package for Social Science
