# Supplementary material for: Sexual violence and associated factors among women of reproductive age in Rwanda: a 2020 nationwide cross-sectional survey
Source: Arch Public Health. 2023 Jun 19;81:112. doi: 10.1186/s13690-023-01109-z (PMC10278278; doi:10.1186/s13690-023-01109-z)
Supplement: Supplementary file 1 — Supplementary Material 1 [file 13690_2023_1109_MOESM1_ESM.docx]

**Sexual violence and associated factors among women of reproductive age in Rwanda**

**What is known?**

Sexual violence is one of the commonest forms of violence experienced by women and a major public health concern. Sexual violence negatively impacts both the victims and their families with effects like contracting sexually transmitted infections including Human Immunodeficiency Syndrome, unwanted pregnancies, stigmatization, and depression among others. There is a paucity of studies about sexual violence and its predictors among women of reproductive age in Rwanda. Existing few studies have focused on pregnant women and female sex workers.

**What does the study add?**

Our study revealed a substantial prevalence of sexual violence among women of reproductive age in Rwanda which is higher than the overall worldwide prevalence. Social demographic factors such as justified beating, health insurance, sex of household head, healthcare decision-making, partner's education, and husband’s frequency of getting drunk were found to be the key determinants of sexual violence among women of reproductive age compared to other factors. Additionally, the study found that women who lived in households headed by males were less likely to face sexual violence than those who lived in female-headed households.

**What the implications are for clinical practice, public health and/or research?**

This is the first nationwide study which used the most recent national dataset hence the findings can be generalized to all women in Rwanda. Therefore, the findings will be used to inform policy such as the one related to demystifying negative culturally-rooted beliefs favoring sexual violence like justified beating as well as increasing efforts to promote women's empowerment and healthcare access.
